# Comparison of Enamel Morphologic Characteristics after Conditioning with Various Combinations of Acid Etchant and Er:YAG Laser in Bonding and Rebonding Procedures: A SEM Analysis

**Published:** 2017-05

**Authors:** Mohammad Sadegh Ahmad Akhoundi, Ardavan Etemadi, Maryam Nasiri, Elahe Soltanmohamadi Borujeni

**Affiliations:** 1 Professor, Dental Research Center, Dentistry Research Institute, Tehran University of Medical Sciences, Tehran, Iran; Department of Orthodontics, School of Dentistry, Tehran University of Medical Sciences, Tehran, Iran; 2 Assistant Professor, Laser Research Center of Dentistry, Dentistry Research Institute, Tehran University of Medical Sciences, Tehran, Iran; Department of Periodontics, Dental Branch, Islamic Azad University, Tehran, Iran; 3 Postgraduate Student, Department of Orthodontics, School of Dentistry, Tehran University of Medical Sciences, Tehran, Iran

**Keywords:** Scanning Electron Microscopy, Orthodontics, Laser

## Abstract

**Objectives:**

Many studies have evaluated re-etched enamel by using scanning electron microscopy (SEM); however, there is no evidence regarding the use of Erbium-doped yttrium aluminium garnet (Er:YAG) laser at primary and secondary bonding instead of acid etching with regards to enamel surface changes. The purpose of the present study was to determine that whether or not the methods of primary and secondary enamel preparation affect enamel characteristics after rebonding, by using SEM analysis.

**Materials and Methods:**

Twelve freshly extracted premolars were divided into 4 groups. The samples in each group were conditioned by acid etchant or Er:YAG laser at primary conditioning, according to the instructions. Afterwards, they were bonded with orthodontic brackets. After debonding, the samples were prepared for second conditioning. Also, two samples were conditioned only once with acid etchant or laser, to compare enamel morphology changes with those after re-etching. Finally, buccal enamel surfaces were evaluated using SEM.

**Results:**

Enamel etching patterns were observed in the samples which had been acid-conditioned at first or at both conditionings. The samples irradiated by Er:YAG laser showed amorphous and irregular surfaces, with no signs of typical etching patterns. A large deep gap was seen in one of the samples irradiated with laser at primary and secondary conditionings, which might have penetrated the underling layers of enamel and dentin.

**Conclusions:**

Enamel surface preparation with Er:YAG laser produces irregular and indistinct morphologic changes, completely different from those produced after acid etching at both conditioning and reconditioning. Therefore, it is recommended to use this laser with caution to avoid permanent enamel damage.

## INTRODUCTION

Direct bonding to enamel is one of the important parameters in orthodontics. Since a major part of success in bonding is related to the enamel preparation method, familiarity with enamel characteristics is essential. Currently, the use of 37% phosphoric acid is the standard protocol for enamel conditioning [1]. In general, the changes in the enamel surface after acid etching are categorized into three types [2]. In type 1, a honeycomb appearance is produced, since prism core material is preferentially removed, leaving the prism peripherally intact. In type 2, a cobblestone effect is produced by preferential dissolution of peripheral regions of the prisms, leaving the prism cores intact. Etching pattern type 3 has areas corresponding to both types 1 and 2. However, due to some major disadvantages of acid-etch technique, such as removal of superficial enamel, various etching depths, high sensitivity to water or saliva contamination, etc., lasers such as Erbium-doped yttrium aluminium garnet (Er:YAG) are considered as an alternative for enamel conditioning, since this laser seems to be effective in removal of hard dental tissues with only minor side effects, such as thermal damage [3]. Since the side effects and disadvantages of acid etching jeopardize the bonding quality, it is necessary to identify the effects of alternative enamel conditioning methods with regards to enamel morphology and bond strength. Also, it should be noted that there are many conflicts about these parameters in different articles. In terms of shear bond strength, the results of many articles have confirmed that Er:YAG laser can be an appropriate alternative for acid etching [4–9]; however, some other researchers have rejected this hypothesis [10–13]. Some studies have evaluated the enamel morphological characteristics through scanning electron microscopy (SEM) after enamel conditioning using Er:YAG laser. In 2011, Brauchli et al investigated the effect of Er:YAG and carbon dioxide (CO2) lasers on enamel surface structure of bovine incisors [1]. They concluded that the use of Er:YAG laser resulted in a surface similar to type 3 etching pattern. Micro-fissures were observed on the enamel surface, which indicate that Er:YAG laser is inappropriate for enamel conditioning. Subsurface fissures beyond normal resin penetration depth after Er:YAG laser ablation were also reported by Dunn et al [14]. In 2015, Sawan et al found that Er:YAG laser-ablated surfaces showed higher number of craters [15]. In contrast, Keller and Hibst observed encouraging results after using Er:YAG laser, with only minimal damage of the surrounding tissue [16]. Considering the increasing number of orthodontic patients, and need for bracket repositioning in some cases, or inadvertent bracket debonding during treatment, standardization of rebonding procedures such as secondary enamel conditioning is necessary to maximize the efficiency of treatment and reduce costs. Considering the potential disadvantages of acid-etch technique and proposed benefits of Er:YAG laser as an alternative modality in bonding and rebonding, it seems logical to compare different enamel conditioners for bonding and rebonding in terms of shear bond strength, remnant adhesives, mode of bonding failure and enamel characteristics. In this study, by using SEM, the enamel morphology after irradiation with Er:YAG laser was evaluated and compared to that after the use of conventional acid-etch method. We also determined that whether the method of primary and secondary enamel preparation (by acid etchant and Er:YAG laser) affects enamel characteristics in rebonding.

## MATERIALS AND METHODS

In this in-vitro study, two modalities (37% phosphoric acid and Er:YAG laser) were used for enamel conditioning. Fourteen freshly extracted premolars were used in this study. All the teeth were thoroughly cleaned under tap water and were immersed in 0.5% chloramine solution for 48 hours for disinfection. Afterwards, they were stored in distilled water at room temperature.

Prior to bonding, buccal enamel surfaces were polished using a low-speed handpiece, rubber cup, and pumice for 10 seconds, and were thoroughly washed for 5 seconds. Afterwards, 12 teeth were randomly divided into 4 groups:

Group 1. The teeth were conditioned with 37% phosphoric acid gel at primary and secondary bonding processes.
Group 2. The teeth were conditioned with 37% phosphoric acid at first bonding, and with Er:YAG laser at second bonding.
Group 3. The teeth were conditioned with Er:YAG laser at first bonding, and with 37% phosphoric acid at second bonding.
Group 4. The teeth were conditioned with Er:YAG laser at primary and secondary bonding processes.

In groups 1 and 2, the enamel was etched with 37% phosphoric acid gel for 15 seconds (3M Unitek, Monrovia, CA, USA), rinsed for 10 seconds and air-dried. If a tooth does not show a frosty and chalky appearance after etching, the process can be repeated. In the current study, all the teeth showed this appearance after the first-time etching; therefore, there is no risk of bias regarding this issue. Etching was performed at the midpoint of the buccal surface, determined with an electronic gauge, equal to the size of a bracket. In groups 3 and 4, the midpoints of buccal surfaces were conditioned by Er:YAG laser (PLUSER, Doctor Smile, LAMBDA SPA, Italy), with the following specifications: power: 2W, energy: 200mJ, frequency: 10Hz, time: 10sec, distance: 2mm, water: 70%, air: 90%. After exposing the enamel surface to laser, a white, frosty and porous appearance was seen due to ablation of enamel structure.

After etching, the teeth were bonded at the midpoint of the anatomic crown using 12 premolar brackets (Dentaurum, Germany), by applying a special primer (Transbond XT, 3M Unitek, Monrovia, CA, USA) and adhesive (Transbond XT, 3M Unitek, Monrovia, CA, USA). After adding a thin layer of the primer to the buccal surface, the area was cured for 5 seconds. Then, the brackets were placed on the midpoint of the anatomic crowns, and each surface was cured by a halogen LED curing device (LED.D Curing Light, Guilin Woodpecker Medical Instrument Co., Ltd. China; wavelength: 440–480nm) for 40 seconds (10 seconds for each side). At this point, the brackets were debonded using debonding pliers, and the remaining composites were removed from the buccal surface by the use of a tungsten carbide finishing bur and a low-speed handpiece. The teeth were prepared for second enamel conditioning according to the instructions. The two remained teeth were conditioned with acid etchant or Er:YAG laser (1 by acid, and 1 by laser) only once, and then they were evaluated under SEM to detect the enamel changes after first conditioning.

Finally, the samples were sent to a special laboratory for SEM analysis by a blinded operator. The specimens were fixed onto a specimen holder, and then they were dehydrated and sputter-coated with a layer of gold. A scanning electron microscope (TESCAN, Czech Republic) was employed to observe the surface changes produced by laser irradiation and phosphoric acid, at magnifications of 500, 2000, 5000, and 10000× to better visualize the details. The present study was performed to detect the changes in the enamel structure after using the mentioned methods. Shear bond strength has not been assessed in any of the stages of bonding.

## RESULTS

**Fig. 1:**
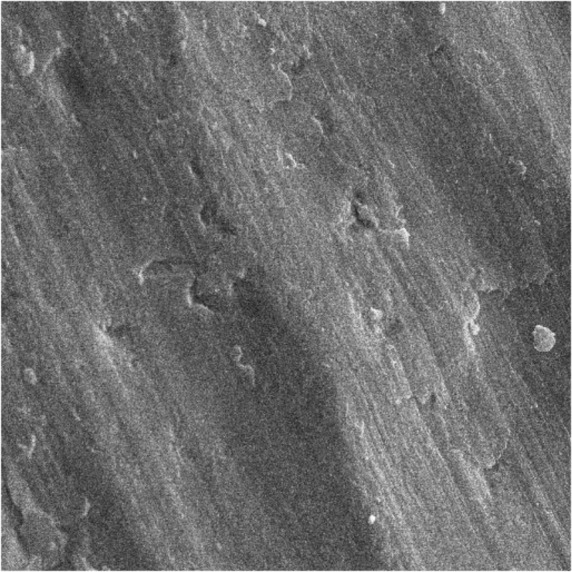
Intact enamel structure

**Fig. 2:**
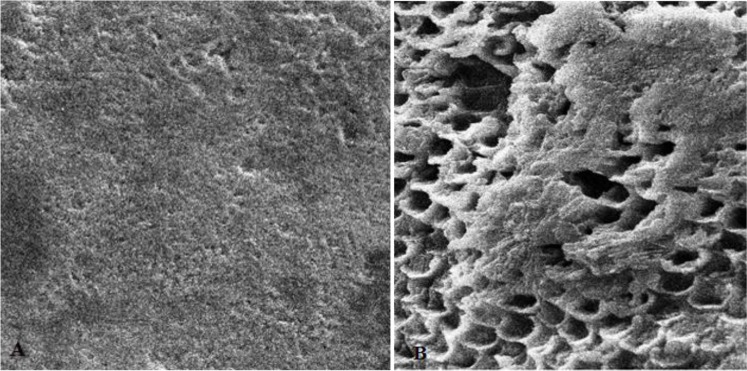
Enamel surface after primary conditioning by acid etching. A: Magnification: 500×, B: Magnification: 2000×

**Fig. 3:**
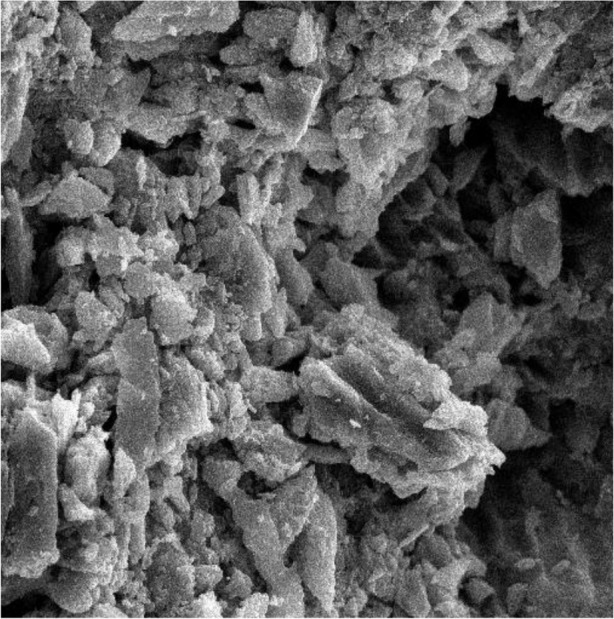
Enamel surface after first conditioning with laser (magnification: 2000×)

**Fig. 4:**
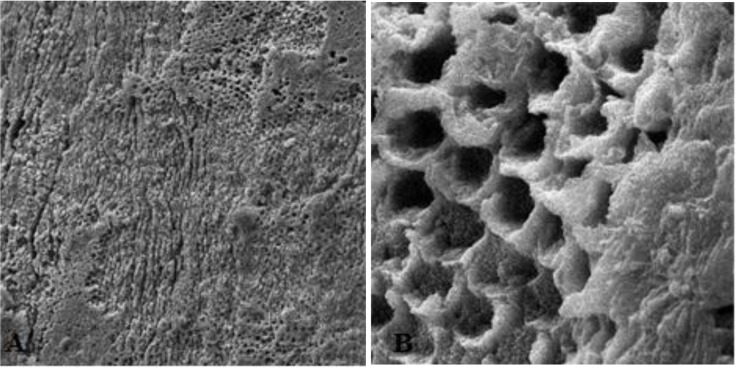
Enamel etching pattern type 1 (honeycomb appearance). A: Magnification: 500×, B: Magnification: 5000×

**Fig. 5:**
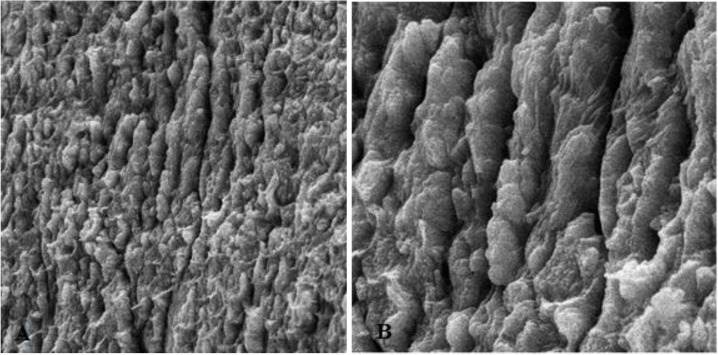
Enamel etching pattern type 2. A: Magnification: 2000×, B: Magnification: 5000×

**Fig. 6:**
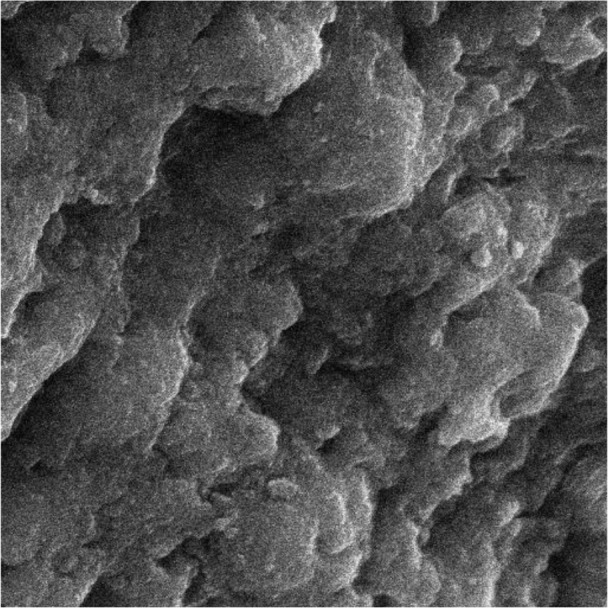
Amorphous areas in etched enamel surface

**Fig. 7:**
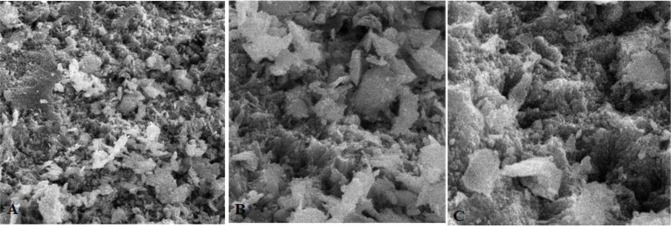
Enamel characteristics in group 2 after second conditioning, A: Magnification: 2000×, B: Magnification: 5000×, C: Magnification: 10000×

**Fig. 8:**
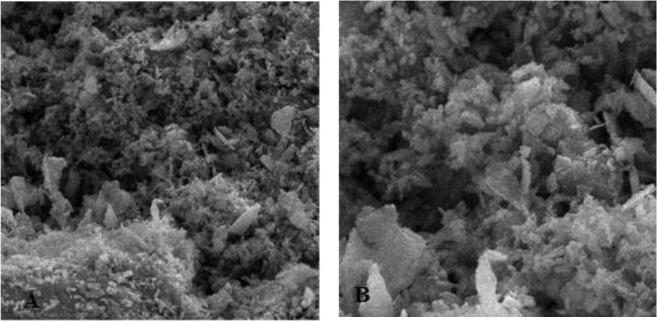
Enamel characteristics in group 3 after second conditioning. A: Magnification: 5000×, B: Magnification: 10000×

**Fig. 9:**
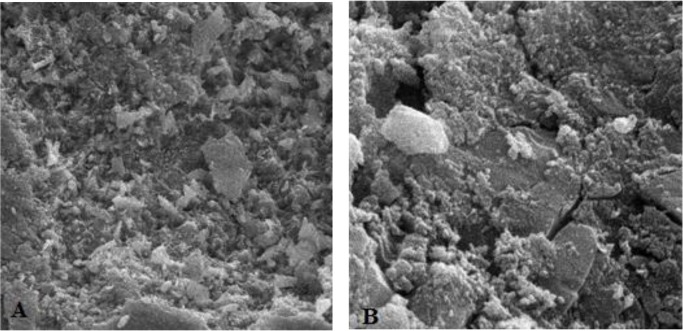
Enamel characteristics in group 3 after second conditioning. A: Magnification: 2000×, B: Magnification: 5000×

First, the intact enamel surface was observed at 500× magnification. The surface was smooth with some micro-cracks and tears in different regions, which were not clinically evident (Fig. 1). The enamel surface after primary acid etching showed a typical appearance which was comparable with type 1 etching pattern in almost all areas. Some parts of the intact enamel showed micro-cracks that did not seem to be extensive or deep (Fig. 2). We could not find any regions with type 2 or 3 etching pattern, but some amorphous areas were evident with mixed and irregular structure. In contrast to this distinct enamel morphology in the acid-etched sample, the lased sample showed a completely irregular and coarse structure with no obvious patterns in any region that could be explained by micro-explosions occurred due to laser irradiation, which have had a destructive effect on the enamel (Fig. 3). Distinct morphological surface changes caused by acid etching were also clearly demonstrated in the teeth conditioned with phosphoric acid at both conditionings (group 1). The honeycomb appearance (pattern 1), and enamel core materials with dissolution of peripheries (pattern 2) were seen in association with enamel cracks and tears in some areas (Fig. 4 and 5). We also observed few areas of amorphous appearance (Fig. 6). These findings were similar to those in the acid-etched sample. It seems that typical changes occurred in the enamel structure at both etching and re-etching processes. The extent of the amorphous areas was minimum in these teeth, and severe enamel damages or deep scrapes were not observed in any region. In contrast, the enamel surface in all other groups showed an amorphous appearance with no distinct enamel etching pattern, which was comparable to the appearance of the sample etched only once with Er:YAG laser. The prisms and their peripheries were indistinguishable, and the enamel structure was disorderly and mixed. No enamel cracks were observed in any of these groups, and there were significantly fewer porosities in comparison to the acid-etched group. Figures 7 to 9 show enamel morphology in groups 2 and 3, respectively.

**Fig. 10:**
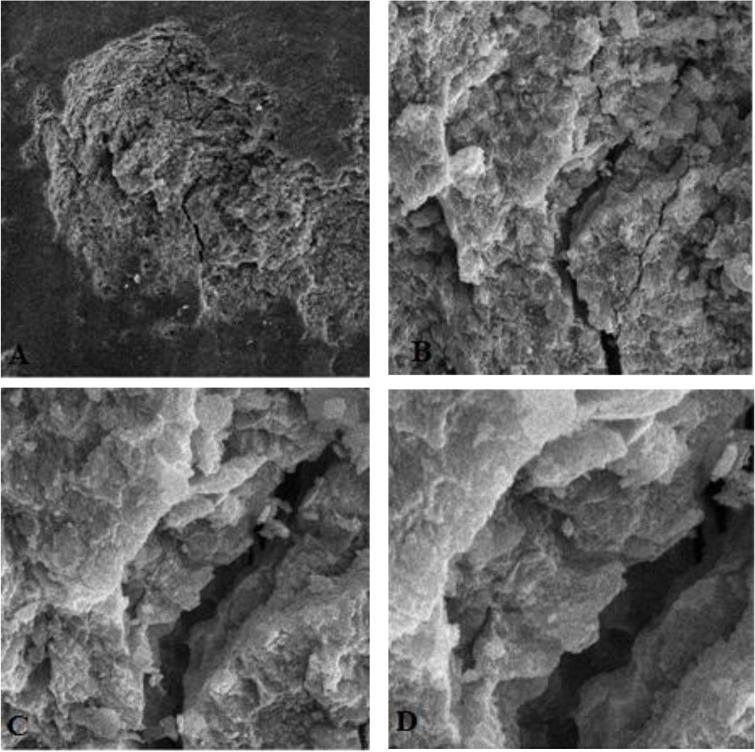
A large gap in enamel in one of the samples of group 4. A: Magnification: 500×, B: Magnification: 2000×, C: Magnification: 5000×, D: Magnification: 10000×

We also found that in one of the samples of group 4, which had been conditioned by laser at both stages, a large deep gap was created in enamel, which was not seen in other teeth, and seemed to have penetrated deeply into the inferior layers of enamel and maybe dentin (Fig. 10). It can be understood from the above information regarding enamel characteristics, that conventional acid etching is more suitable for etching and re-etching of enamel in comparison with Er:YAG laser.

## DISCUSSION

In the present article, we compared the differences in enamel morphological features after the use of conventional acid etchant and Er:YAG laser in bonding and rebonding. We also evaluated the effects of the enamel conditioning method used at primary bonding on the enamel morphology after secondary conditioning. SEM helped us to estimate the amount of enamel destruction. The typical etching patterns were observed only in the samples which had been conditioned with acid etchant, or conditioned and reconditioned with acid etchant, without the use of laser in any of the stages of bonding. Other teeth showed amorphous and mixed enamel structure, without any distinct pattern. There is a lack of evidence in the literature regarding the differences of the two methods in rebonding, and regarding their interactions; however, some articles have investigated the differences of these two methods in primary bonding. As mentioned in the introduction, different surface structures have been described after conditioning with Er:YAG laser. In a study by Hess, this laser with predetermined specifications produced an irregular and roughened pattern with micro-cracks on the surface [17]. Sasaki et al [18] found that the acid-etched group had a more homogeneous etching pattern on the treated surfaces, which confirms our findings; however, it was stated that the specimens conditioned only with Er:YAG laser showed areas of ablation, which contained nonlased enamel. They concluded that irradiation with Er:YAG laser followed by acid etching resulted in a more homogeneous surface pattern, compared to the surfaces treated only with laser. The results of our study at both steps of enamel conditioning were similar to those of the mentioned study. The lased samples showed a non-homogenous structure, when compared with the acid-etched specimens at both etching and re-etching. Martinez-Insua et al also stated that the enamel and dentin surfaces prepared by Er:YAG laser show extensive subsurface fissuring that is unsuitable for adhesion [13].

In 2011, Brauchli et al [1] concluded that the use of Er:YAG laser resulted in surfaces similar to type 3 etching pattern. They stated that surface morphology with a network of micro-fissures can raise concerns about the use of this laser. Micro-fissures were also detected in two other investigations [5,13]. We found a large gap in one of the samples in group 4, which had been conditioned only by laser. The main reason of this gap formation is unknown.

As we surveyed the enamel surface morphology, we could not measure the depth of the gap, but it seemed to have penetrated into the inferior layers of enamel and maybe dentin. Since similar findings were not observed in other samples of this group, the clinical importance of this gap remained questionable, and more samples conditioned with laser at both stages should be evaluated to determine whether there are any destructive unfavorable changes in the deep layers of enamel.

Sawan et al [15] stated that laser-ablated surfaces show the formation of craters, and have suitable enamel roughness. We did not observe enamel craters, but surface roughness seemed to be acceptable in most situations. Some authors concluded that surface roughness after laser irradiation is similar to that after conventional acid etching. Keller and Hibst confirmed favorable changes in enamel after using Er:YAG laser, with no or minor enamel damages [16]. Sawan et al believed that Er:YAG laser etching can replace acid etching, with similar impacts on enamel and without the negative effects of phosphoric acid etching [15], which is in contrast to our results. In terms of enamel structure and morphology, Er:YAG laser cannot be a good replacement for conventional acid etching. The mechanism of tissue removal by laser, unlike acid etching, is not demineralization, and the enamel is ablated due to laser energy. It is possible that the heterogeneous enamel structure in groups in which the samples had been lased at first or second bonding procedures, is due to this ablation process. Since the changes in enamel after re-etching were similar to the ones after first conditioning, it seems logical to conclude that the enamel preparation method used for primary conditioning affects enamel characteristics after re-conditioning, with favorable results observed in the samples conditioned with acid etchant at primary, or at primary and secondary conditionings. The enamel structure is lost after conditioning by Er:YAG laser, and an amorphous and irregular structure remains, with possibility of enamel gaps and damages in some areas. Further investigations are recommended to clearly determine the effects of Er:YAG laser on rebonding under different conditions, especially on the potential changes in the underlying layers of enamel and dentin.

## CONCLUSION

The enamel structure was more homogenous in control group compared to the groups conditioned by laser at least once during first or second conditioning. It can be concluded that damage to the enamel structure can be more profound when Er:YAG laser is used in any of the stages of bonding. This damage could compromise the results of enamel rebonding, and may adversely affect the shear bond strength.
